# Changing Gastrointestinal Transit Time Alters Microbiome Composition and Bile Acid Metabolism: A Cross‐Over Study in Healthy Volunteers

**DOI:** 10.1111/nmo.70075

**Published:** 2025-05-20

**Authors:** Evette B. M. Hillman, Maximilian Baumgartner, Danielle Carson, Gregory C. A. Amos, Imad Wazir, Haider A. Khan, Malik A. Khan, Sjoerd Rijpkema, Julian R. F. Walters, Elizabeth M. H. Wellington, Ramesh Arasaradnam, Stephen J. Lewis

**Affiliations:** ^1^ Diagnostics, Medicines and Healthcare Products Regulatory Agency London UK; ^2^ School of Life Sciences The University of Warwick Coventry UK; ^3^ Division of Gastroenterology and Hepatology Medical University of Vienna Vienna Austria; ^4^ CeMM Research Center for Molecular Medicine of the Austrian Academy of Sciences Vienna Austria; ^5^ Department of Gastroenterology University Hospital Plymouth Plymouth UK; ^6^ Division of Digestive Diseases Imperial College London London UK; ^7^ Imperial College Healthcare Trust London UK; ^8^ Department of Gastroenterology University Hospitals Coventry & Warwickshire Coventry UK; ^9^ Warwick Medical School The University of Warwick Coventry UK; ^10^ Peninsula Medical School University of Plymouth Plymouth UK

**Keywords:** bile acids metabolism, gastrointestinal homeostasis, loperamide and senna treatment, microbiome, whole‐gut transit‐time (WGTT)

## Abstract

**Background:**

The specific influence of whole gut transit time (WGTT) on microbiome dynamics and bile acid metabolism remains unclear, despite links between changes in WGTT and certain gastrointestinal disorders. Our investigation aimed to determine the impact of WGTT changes on the composition of the fecal microbiome and bile acid profile.

**Methods:**

Healthy volunteers (*n* = 18) received loperamide, to decrease bowel movement frequency, and senna, a laxative, each over a 6‐day period, in a randomized sequence, with a minimum 16‐day interval between each treatment. Stool samples were analyzed for microbiome by shotgun sequencing and bile acid composition determined with high‐performance liquid chromatography coupled to tandem mass spectrometry. Sera were examined for markers of bile acid synthesis.

**Key Results:**

Senna or loperamide decreased or increased WGTT, respectively. Treatment altered stool characteristics, bowel movement frequency, and stool weight. The senna‐treated group had increased primary and secondary fecal bile acids; serum levels of fibroblast growth factor 19 were significantly reduced. Increasing WGTT with loperamide led to an increase in bile salt hydrolase genes, along with elevated bacterial species richness (*p* = 0.04). Thirty‐six species exhibiting significant differences were identified, several of which have notable implications for gut health. WGTT displayed negative correlations with total primary (particularly chenodeoxycholic acid) and secondary bile acids (ursodeoxycholic acid and glycochenodeoxycholic acid). Treatment‐induced changes in microbiome composition and bile acid metabolism reverted back to baseline within 16 days.

**Conclusion:**

Whole gut transit time changes significantly affect fecal microbiome composition and function, as well as bile acid composition and synthesis in healthy subjects. This consideration is likely to have long‐term implications.

## Graphical abstract

Alterations in whole gut transit time (WGTT) significantly impact microbiome composition and bile acid metabolism. Using senna and loperamide, we show that WGTT changes influence bacterial diversity, bile acid profiles, and fibroblast growth factor 19, with effects reversing after 16 days—highlighting the dynamic gut‐microbiome‐bile acid interplay.

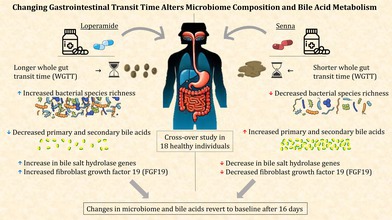

**Abbreviations**
BAD: bile acid diarrhea
BAI: bile acid‐inducible
BL‐L: baseline before loperamide
BL‐S: baseline before Senna
BSH: bile salt hydrolase
C4: 7α‐hydroxy‐4‐cholesten‐3‐one
CA: cholic acid
CDCA: chenodeoxycholic acid
CRC: colorectal cancer
DCA: deoxycholic acid
ELISA: enzyme‐linked immunosorbent assay
FGF19: fibroblast growth factor 19
GCA: glycocholic acid
GCDA: glycochenodeoxycholic acid
GI: gastrointestinal
GUDCA: glycoursodeoxycholic acid
HCA: hyocholic acid
HDCA: hyodeoxycholic acid
HPLC–MS/MS: high‐performance liquid chromatography coupled to tandem mass spectrometry
IBS: irritable bowel syndrome
IDI: inter‐defecatory interval
LCA: lithocholic acid
LC–MS/MS: liquid chromatography–tandem mass spectrometry
MCA: muricholic acid
PCR: polymerase chain reaction
SCFAs: short‐chain fatty acids
TCA: taurocholic acid
TCDCA: taurochenodeoxycholic acid
TUDCA: tauroursodeoxycholic acid
UDCA: ursodeoxycholic acid
WGTT: whole‐gut transit time

**Summary**

- Changes in whole gut transit time (WGTT) are linked to gastrointestinal disorders, but their specific effects on the fecal microbiome and bile acid metabolism are not fully understood.
- WGTT alterations significantly impact the fecal microbiome's composition and function, as well as bile acid profiles, demonstrating notable changes that revert back to ‘normal’ within 16 days.
- Understanding the relationship between WGTT and gut microbiome composition can inform treatments for gastrointestinal disorders, as manipulating transit time may influence gut health through microbiota and bile acid profiles.

## 1 Introduction

The time it takes for food to travel from the oral cavity until it is expelled in stool is known as the whole‐gut transit‐time (WGTT). The reduction or lengthening of gastrointestinal (GI) transit time is associated with numerous human pathologies of varying severity, often as a symptom revealing the underlying condition: Parkinson's disease, where sufferers usually experience constipation [1], irritable bowel syndrome (IBS) which can present as constipation and/or diarrhea [2] and colorectal cancer (CRC) associated with reduced motility [3].

The gut microbiome is considered central in the development and function of physical and mental health [4, 5]. As the colon harbors the densest number of microbes out of all the organs [6], it is imperative to examine the link between the gut microbiome and the WGTT. Numerous population‐wide studies have established an association between the richness of gut microbiota, diversity, composition, and the speed at which food moves through the intestines [7, 8]. While a correlation between intestinal transit‐time and changes in the microbiome has been observed [8, 9, 10, 11], the causal relationship between the two remains unclear.

Key bacterial metabolites in the human gut include bile acids [12, 13, 14] and short‐chain fatty acids (SCFAs) [15, 16], which play critical roles in a variety of physiological processes. Changes in the bile acid pool have been linked to gut transit time and several diseases including gallstones, CRC and bile acid diarrhea (BAD) [17, 18, 19, 20]. There are two types of bile acids: primary and secondary bile acid. Primary bile acids are synthesized in the liver and in humans are cholic acid (CA) and chenodeoxycholic acid (CDCA). These are conjugated to either taurine or glycine. The secondary bile acids deoxycholic acid (DCA) and lithocholic acid (LCA) are synthesized by bacteria in the human intestine from CA and CDCA respectively. While there are many other secondary bile acids, DCA and LCA are the most abundant in human feces, at 34% and 29%, respectively [21]. In the synthesis of bile acids from cholesterol, a key intermediate is 7α‐hydroxy‐4‐cholesten‐3‐one (C4). Bile acids regulate their own expression through feedback inhibition, which is mediated by the ileal hormone fibroblast growth factor 19 (FGF19). Additionally, SCFAs such as acetate, propionate and butyrate are considered essential biomarkers for evaluating gut health as they improve gut barrier function, boost the immune system, and reduce inflammation [16, 22].

While correlations between WGTT changes and shifts in microbial composition have been established, these offer only limited insights into the impact on gut health. Hence, analysis of microbiome function and metabolites, including bile acids and SCFAs is needed.

To determine how WGTT influences microbiome and bile acid composition independent from disease, we examined the effects of increasing and decreasing WGTT in healthy volunteers using over‐the‐counter medicines. WGTT was increased with loperamide, used to treat diarrhea, while reduced with senna, a laxative. We analyzed fecal microbiome composition with high‐throughput shotgun sequencing, and examined bile acids in stool, and parameters for bile acid metabolism in sera. This randomized cross‐over study in healthy individuals aimed to explore the direct effects of WGTT on the intestinal microbiome, bile acid composition, and metabolism in humans.

## 2 Methods

### 2.1 Volunteer Recruitment

The study included 18 healthy adult volunteers who fulfilled specific eligibility criteria: no history of gut‐related conditions such as IBS, coeliac disease, or inflammatory bowel disease (IBD) within the preceding year, and regular bowel habits. The participant group had a male‐to‐female ratio of 2:1, a median age of 34 years (IQR 28–52), and a median BMI of 25.9 (IQR 23.2–27.6). All participants were omnivorous, nonsmokers, not on medication, and not pregnant, with no notable medical history. Samples were collected between April 2017 and May 2018 at University Hospitals Plymouth. The study was approved by the NHS Health Research Authority (18/NW/0346), and all participants provided written informed consent. Individuals who had used probiotics, antibiotics, or other gut microbiota‐altering agents within the previous 3 months were excluded (Table S1).

### 2.2 Study Design

This is a randomized cross‐over observational study without a predefined primary outcome or specific clinical endpoints. The primary objective was to assess the impact of altered intestinal transit time on the enterohepatic recirculation of bile acids. Additional analyses included evaluating changes in the gut microbiome, bile acid composition, and biomarkers of hepatic bile acid synthesis. It was registered: https://edge.nhs.uk.

**FIGURE 1:**
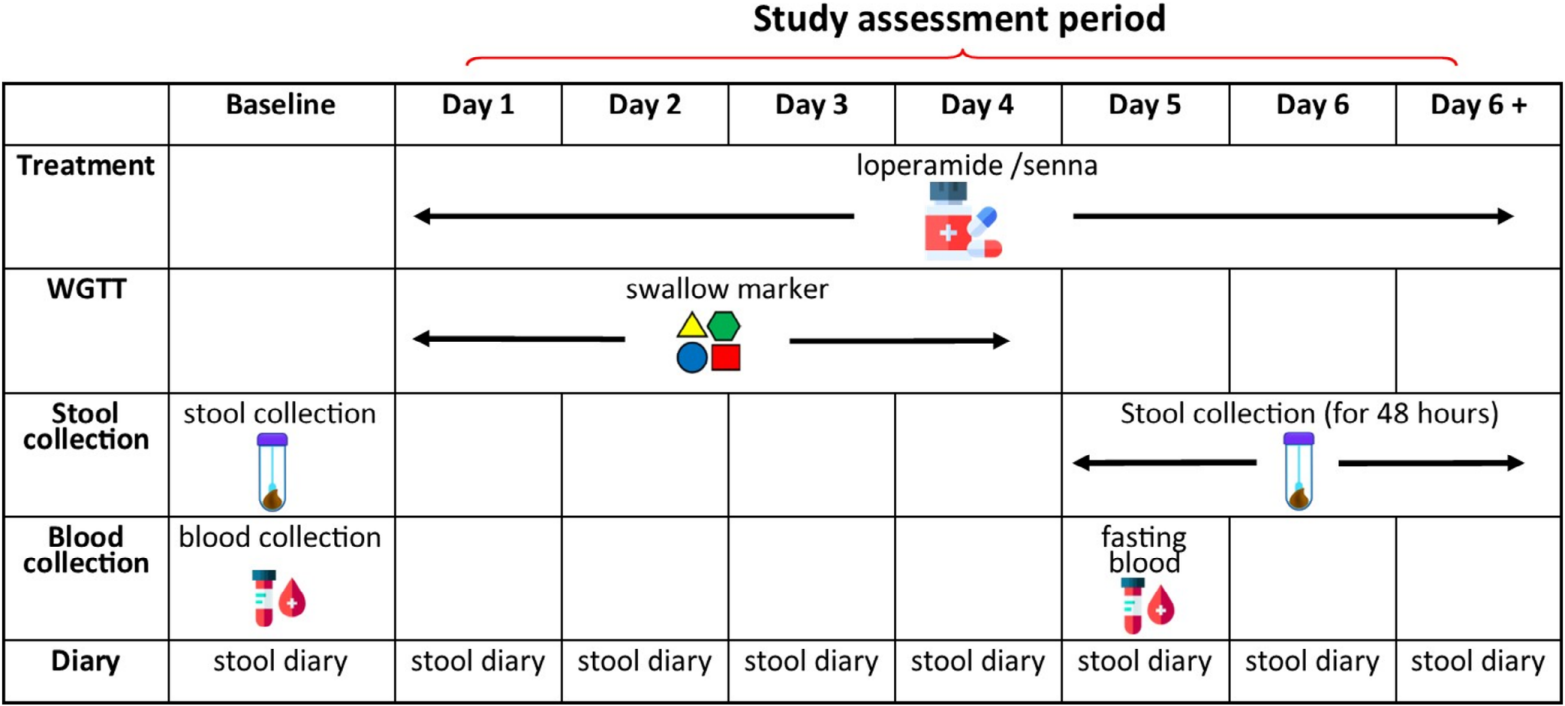
Experimental plan depicting when samples were collected and when medication was taken.

In this study, volunteers underwent four separate assessment periods. A baseline assessment, including fecal and serum sample collection and transit time measurement, was conducted before any drug administration. Following this, participants entered the assessment period, during which they received either senna (Thornton & Ross Ltd., Huddersfield, UK) or loperamide (Tillomed Lab Ltd., St Neots, UK). These drugs were administered in random order and taken at the maximum tolerated dose for 2 days prior to and throughout the assessment period (lasting 5–8 days) see Figure 1. After a washout period of at least 16 days, participants repeated the same assessments with the alternate drug. Volunteers were asked to copy their baseline diets as far as possible for each further assessment period (photocopied initial baseline diary). The dietary intake for each assessment period was tabulated and analyzed using nutritional analysis software DietPlan7 (Forrestfield Software Ltd., Horsham, UK) for total fluids, energy, protein, carbohydrate, and fat [23].

All 18 volunteers participated in the senna treatment phase, with samples successfully collected from all participants. However, only 16 volunteers completed the loperamide phase, resulting in two dropouts. Whole‐gut transit time (WGTT) measurements, bile acid profiling, and microbiome analyses were conducted on all available samples.

### 2.3 Whole Gut Transit‐Time Measurements

Whole‐gut transit‐time was measured by swallowing capsules containing 20 radio‐opaque markers of different shapes each morning for 4 days. The initial two stools passed at least 24 h following the ingestion of the final set of markers were collected and weighed. The stools were flattened and X‐rayed to calculate the WGTT by counting the number of markers [24]. Volunteers kept a defecation diary recording the date and time of defecation which was used to calculate the inter‐defecatory interval (IDI) [25], as well as the stool form using the Bristol Stool Form Scale [24]. After X‐raying, the stool samples were homogenized using a stomacher (Seward Ltd., Worthing, UK) and stored in aliquots at ≤ −70°C for subsequent analysis.

### 2.4 The Microbiome

DNA was purified using DNeasy PowerSoil Kit (Qiagen) from 0.2 g of stool for downstream microbiota and functional genetic analysis. Extraction was carried out according to the manufacturer's protocol. Library preparation for shotgun sequencing was performed using Illumina DNA Prep. Sequencing was carried out on a Nextseq 500 platform (Illumina) using the NextSeq 500/550 v2.5 Kits (Illumina) yielding paired end 150 bp reads with a sequencing depth of around 12.5 million paired‐end reads per sample. Adapter‐trimming, quality‐related trimming, quality filtering, and length filtering were carried out using the program BBDuk which is part of BBTools [26]. MetaPhlAn3 and HUMAnN3 were used from the bioBakery tools to profile the composition of microbial community and the abundance of microbial functional genes respectively [27, 28].

### 2.5 Bile Acid Analysis

The fecal bile acids were measured using high‐performance liquid chromatography coupled to tandem mass spectrometry (HPLC‐MS/MS) as previously described [29]. The following primary bile acids were analyzed: CDCA and CA, their taurine and glycine conjugates (taurochenodeoxycholic acid (TCDCA), taurocholic acid (TCA), glycochenodeoxycholic acid (GCDCA) and glycocholic acid (GCA)), and their sulfated forms: chenodeoxycholic acid 3‐sulfate (CDCA‐3S) and cholic acid 3‐sulfate (CA‐3S). Secondary bile acids LCA, DCA and UDCA conjugated to taurine and glycine (taurolithocholic acid (TLCA), glycolithocholic acid (GLCA), taurodeoxycholic acid (TDCA), glycodeoxycholic acid (GDCA), tauroursodeoxycholic acid (TUDCA), glycoursodeoxycholic acid (GUDCA)) and sulfated (lithocholic acid 3‐sulfate (LCA‐3S), taurolithocholic acid 3‐sulfate (TLCA‐3S), glycolithocholic acid 3‐sulfate (GLA‐3S), deoxycholic acid 3‐sulfate (DCA‐3S), ursodeoxycholic acid 3‐sulfate (UDCA‐3S), tauroursodeoxycholic acid 3‐sulfate (TUDCA‐3S), glycoursodeoxycholic acid 3‐sulfate (GUDCA‐3S) as well as hyocholic acid (HCA), hyodeoxycholic acid (HDCA), taurohyodeoxycholic acid (THDCA), and muricholic acid (MCA)) were also analyzed. Both total primary and secondary bile acids are presented as nmol/g of stool and total nmol/day.

### 2.6 Serological Markers of Bile Acid Synthesis

Fasting serum was collected on the morning of day five after an overnight fast (from midnight) 10 mL of whole blood was taken, and serum was analyzed for FGF19 and C4. C4 was extracted, then purified and measured by liquid chromatography–tandem mass spectrometry (LC–MS/MS) using the method of Honda et al. (2007) [30]. Serum FGF19 was measured using Human FGF‐19 Quantikine ELISA kit following manufacturer's instructions (Bio‐Techne, Abingdon, UK).

### 2.7 Statistical Methods

Being an observational study, sample size was determined by the need to encompass a reasonable range of clinical response times to treatment, as measured by defecatory frequency, drawing from previous studies by Lewis et al. (1997) [24].

To assess changes upon treatment, differences were calculated by normalizing to corresponding baseline value (prefix ‘delta’). WGTT, stool form, IDI, stool weight and bile acid measurements were analyzed using nonparametric paired Wilcoxon test and the unpaired Mann–Whitney test, with results expressed as medians with 95% confidence interval (CI) unless otherwise stated. Alpha diversity was analyzed using Mann–Whitney test and adjusted the *p* values using the Bonferroni correction. Beta diversity was analyzed using a permutational multivariate analysis of variance (PERMANOVA) using the adonis2 function in the vegan package to test for significant differences in microbial community composition. Nonmetric multidimensional scaling (NMDS) ordination was performed on the UniFrac distance matrix to visualize differences in microbial composition among samples. A species comparative analysis was performed using DESeq2 package in R [31]. Wald test was applied to obtain a *p‐value* and Benjamini and Hochberg (BH) (1995) method adjusted *p* values. FGF19 and bile salt hydrolase (*bsh*) gene abundance measurements were analyzed with paired *t*‐tests. Nonparametric Spearman's correlations were performed. Significant adjusted *p*‐values were calculated using the BH method.

All data were analyzed, and graphs plotted using packages in R environment [32] and Prism 9 (GraphPad Software, San Diego, California USA, www.graphpad.com). R packages included phyloseq, vegan, DESeq2 and ggplot2.

## 3 Results

### 3.1 Whole Gut Transit‐Time Measurements

Whole‐gut transit time, inter‐defecatory interval (IDI), stool form (assessed using the Bristol Stool Form Scale) and stool weight were employed as indicators of GI transit dynamics to corroborate the intended outcomes: deceleration of gut transit by loperamide and acceleration of gut transit by senna (Figure S1A–D). Measurements were normalized to corresponding baseline values (shown as ‘delta’) and then the difference was compared between the two treatment groups. Significant differences were detected in the WGTT (*p* = 0.0134), IDI (*p* = 0.0006), stool form (*p* = 0.0002), and stool weight (*p* = 0.0205) between the delta loperamide and delta senna groups. The median WGTT and IDI time in the delta loperamide group were significantly increased compared to delta senna (14.54 vs. −6.14 and 5.8 vs. −3.94 respectively). Stool form, and stool weight per day were both significantly reduced in delta loperamide in contrast to delta senna (median −0.76 vs. 0.71 and −27.63 vs. 85.40, respectively).

### 3.2 Diversity Analysis

**FIGURE 2:**
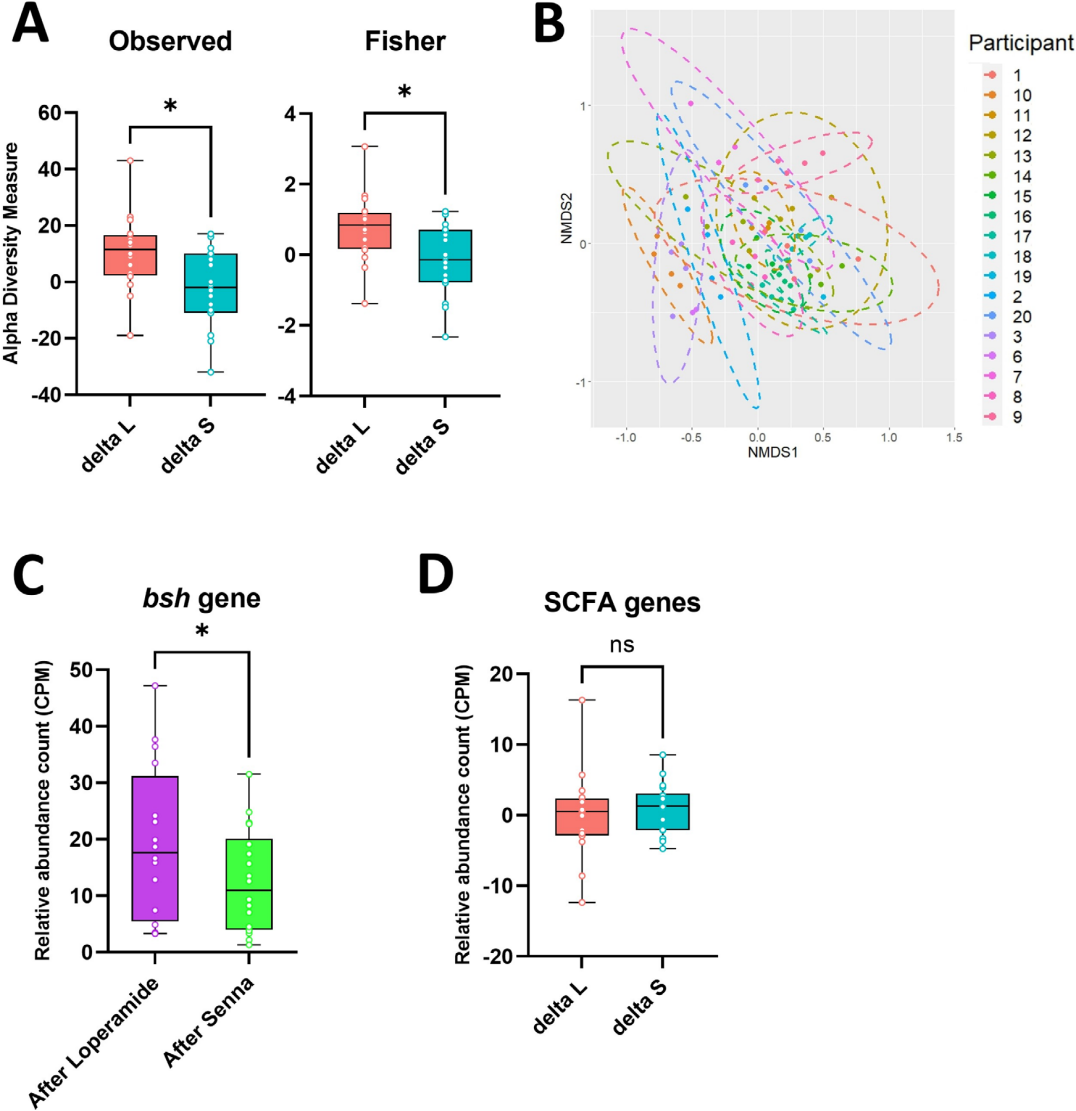
Comparative analysis of microbial diversity, participant clustering, and functional gene abundance in response to loperamide and senna treatment. (A) Species richness across sample types. Observed and Fisher alpha diversity indices of each sample: delta loperamide (delta_L) *n* = 16 and delta senna (delta_S) *n* = 18. (B) Beta‐diversity of participants. Bacterial clustering by participants. Each color represents a difference study participant which comprises of 4 points signifying the 4 samples (Baseline_L, Loperamide, Baseline_S and senna). Nonmetric multidimensional scaling (NMDS) plot with Bray–Curtis dissimilarity distances. Plot ellipses represent the 95% confidence regions for group clusters. *p*‐values calculated unweighted UniFrac test were highly significant *p* < 0.001. (C, D) Microbial Functional Genes. Relative abundance (CPM, copies per million) of (C) *bsh*, (*bile salt hydrolase*) genes after delta taking loperamide (*n* = 16) and senna (*n* = 18) drug and (D) short‐chain fatty acid (SCFA) genes: Delta loperamide (delta_L) *n* = 16 and delta senna (delta_S) *n* = 18. The box‐and‐whisker plots show the medians (horizontal lines), interquartile range (boxes), and maximum and minimum points (whiskers). Differences were assessed with the Wilcoxon test *p ≤ 0.05, ns, not significant.

The prolongation of WGTT with loperamide resulted in a notable and significant increase in species richness compared to the effect of delta senna which accelerated the WGTT Figure 2A. No significant difference was observed in the beta diversity between the sample groups (Baseline_L, Loperamide, Baseline_S and Senna, Figure S2), or when delta loperamide and delta senna were compared. Beta diversity was examined between the different participants (Figure 2B). The samples analyzed from the same individuals congregated together and it was found that there was a statistical significance (*p* = 0.001) in species present between the individuals, suggesting a heterogeneous interpatient microbiome composition.

### 3.3 Functional Microbial Genes

A functional analysis of the microbiota was performed to account for redundancy which cannot be detected by taxonomical studies alone. The relative abundance of *bsh* genes in fecal samples increased after loperamide compared to senna (Figure 2C). Bile salt hydrolases deconjugate bile acids yielding unconjugated bile acids, which is the first step of secondary bile acid biosynthesis. However, no difference was detected in *bsh* genes between the delta loperamide and delta senna groups. In addition, no significant difference was detected between the baselines. No differences were observed in the relative abundance of the genes within the *bile acid‐inducible* (bai) operon—specifically *baiA*, *baiB*, *baiCD*, *baiE*, *baiF*, and *baiN*—which are involved in dehydroxylation and dehydrogenation processes crucial for secondary bile acid synthesis. The *hdhA* gene, although not part of the bai operon, also showed no notable change in relative abundance.

An aggregate analysis of bacterial genes contributing to the synthesis of SCFAs revealed no significant difference in levels between the delta loperamide group and the delta senna group (Figure 2D). Individually, the genes *acetate kinase* (*ackA*), *phosphate acetyltransferase* (*pta*), *phosphate butyryltransferase* (*ptb*), *butyrate kinase* (*buk*), and *propionate CoA‐transferase* (*pct*) implicated in SCFA formation, were also analyzed, and revealed statistically significant (*p* < 0.05) elevated levels of SCFA gene *pta* in the delta loperamide group compared to delta senna group.

### 3.4 Species Comparative Analysis

**FIGURE 3:**
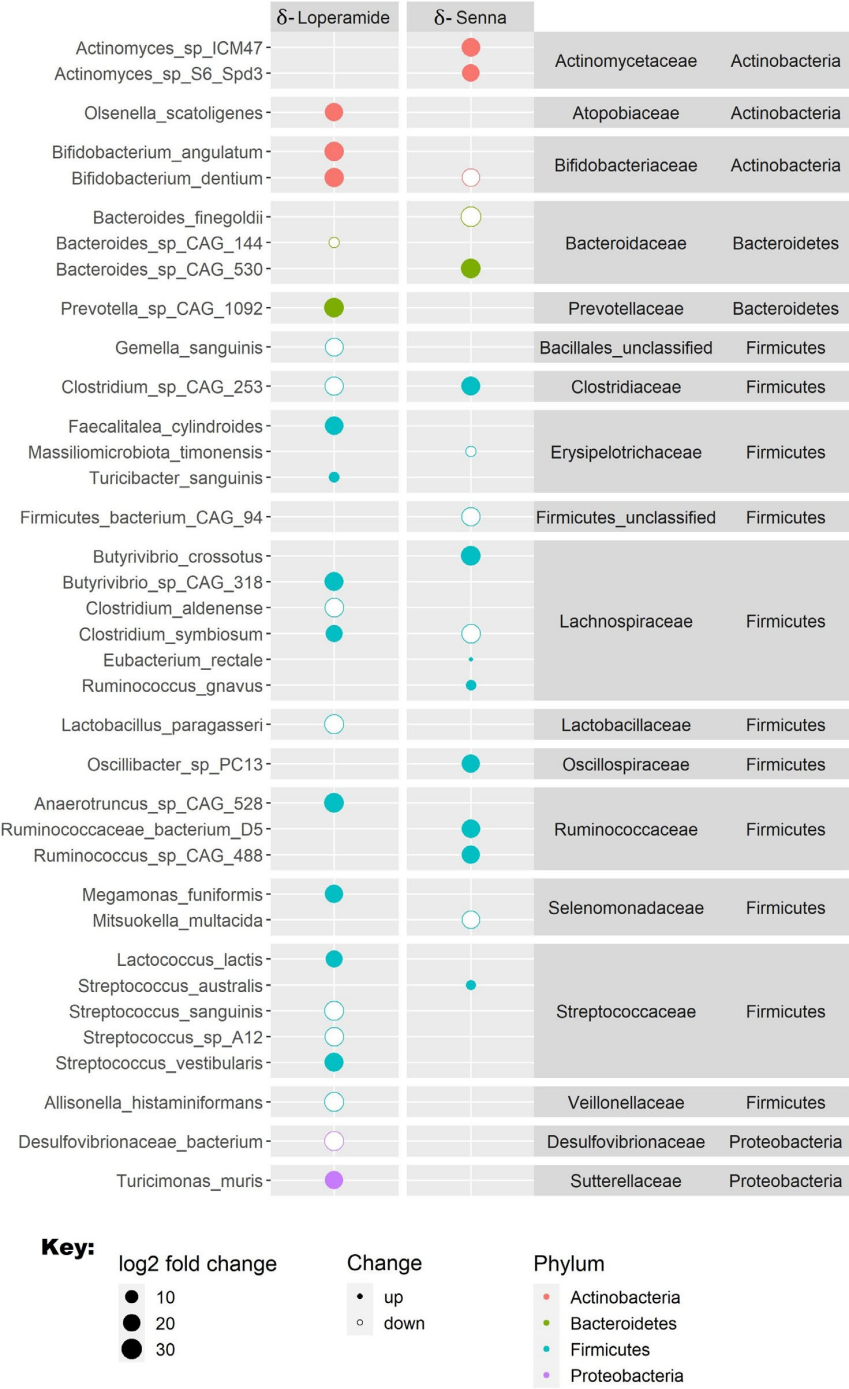
Intestinal species taxonomy changes with loperamide and senna. The log_2_ fold increase or decrease (as determined with DESeq2) between the drug and the corresponding baseline are illustrated. Bacterial taxonomies: Genera and species are shown on the left while family and group are on the right. Only significant findings (*p* < 0.05 after correction for multiple comparisons) are shown. Size of dots signify log_2_ fold‐change, filled dots represent increase in species abundance, empty dots represent decrease in species abundance. Dots are colored based on bacterial phylum. δ‐Loperamide = 16 and δ‐Senna *n* = 18 stool samples.

To further investigate whether there were alterations on microbiome composition, individual species were compared before and after administration of loperamide and senna. Thirty‐six species exhibited significant differences (Figure 3 and Table S2). A number of changes in the abundance of species previously implicated in gut health were observed. *Bifidobacterium dentium* ( *B. dentium* ), *Clostridium symbiosum* ( *C. symbiosum* ), and *Clostridium* sp. CAG 253 were the only species observed to have a significant fold change in both delta loperamide and delta senna. Following loperamide, *B. dentium* and *C. symbiosum* increased by over 28‐log_2_ fold and 21‐log_2_ fold, respectively, compared to baseline, while decreasing by over 23‐log_2_ fold and 25‐log_2_ fold, respectively, following senna. Conversely, *Clostridium* sp. CAG 253 decreased by over 25‐log_2_ fold with loperamide but increased by over 27‐log_2_ fold with senna.

After loperamide, eight bacterial species significantly decreased in stool abundance, including *Allisonella histaminiformans* (26‐log_2_ fold), *Gemella sanguinis* (23‐log_2_ fold), *Lactobacillus paragasseri* (26‐log_2_ fold), *Streptococcus sanguinis* (27‐log_2_ fold), and *Streptococcus* sp. *A12* (26‐log_2_ fold). Ten bacterial species increased including *Anaerotruncus* sp. *CAG 528* (30‐log_2_ fold), *Bifidobacterium angulatum* (28‐log_2_ fold), *Prevotella* sp. *CAG 1092* (29‐log_2_ fold) and *Streptococcus vestibularis* (27‐log_2_ fold). Following senna, four bacterial species decreased, including *Bacteroides finegoldii* (29‐log_2_ fold) and *Firmicutes bacterium CAG 94* (24‐log_2_ fold), while nine species increased, including *Actinomyces* sp. *ICM47* (25‐log_2_ fold), *Bacteroides* sp. CAG 530 (30‐log_2_ fold), Butyrivibrio_crossotus (30‐log_2_ fold), and *Oscillibacter* sp. *PC13* (25‐log_2_ fold), *Ruminococcaceae bacterium* D5 (26‐log_2_ fold), and *Ruminococcus gnavus* ( *R. gnavus* ) (6‐log_2_ fold).

### 3.5 Bile Acid Analysis

Variation in bile acid pool between the volunteers was considerable. Typically, unconjugated bile acids CDCA, CA, LCA, DCA, and UDCA were at the highest concentrations, while conjugated and sulfated bile acids, particularly TUDCA, TLCA‐3S, GLCA, and GUDCA, were the least common and at the lowest concentrations. THDCA, TUDCA‐3S, GUDCA‐3S, HDCA, and MCA were below the limit of detection throughout.

**FIGURE 4:**
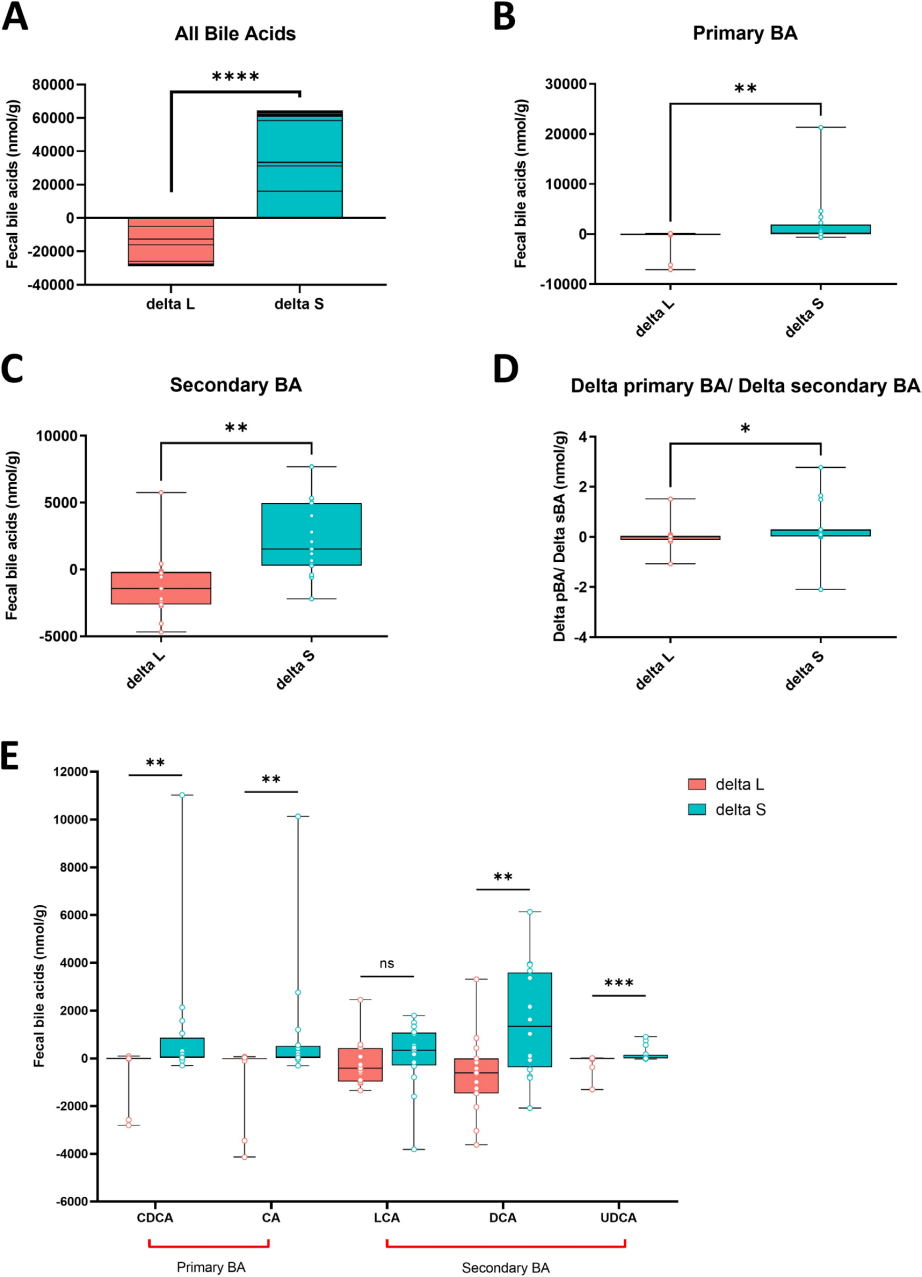
Summary of bile acid analysis response to loperamide and senna. (A) Stacked bar graph showing the total of all individual bile acids and their concentration, (B) primary bile acids, (C) secondary bile acids, (D) the ratio of primary and secondary bile acids and (E) five most abundant bile acids before and after (delta) taking loperamide (*n* = 16) and senna (*n* = 18) drug. Full data are shown in Table S3. Primary bile acids: CA, cholic acid; CDCA, chenodeoxycholic acid. Secondary bile acids: DCA, deoxycholic acid; LCA, lithocholic acid; UDCA, ursodeoxycholic acid. The box‐and‐whisker plots show the medians (horizontal lines), interquartile range (boxes), and maximum and minimum points (whiskers). Differences were assessed with the Wilcoxon test. **p ≤ 0.01, ***p ≤ 0.001, ****p ≤ 0.0001 and ns, not significant.

The sum of the changes in the individual bile acids following treatment (delta loperamide and delta senna) are shown in Figure 4A and individual changes and *p*‐values are shown in Table S3. The concentration of the majority of bile acids decreased in the stool when WGTT increased with loperamide. By contrast, the opposite happened following treatment with senna which accelerated transit, reducing the WGTT. Bile acid concentrations of the delta loperamide group were always lower than delta senna group (*p* < 0.0001) indicating an inverse relationship between bile acids in the stool and the gut transit‐time. A marked reduction in both primary and secondary bile acid concentrations occurred as WGTT lengthened (Figure 4B,C). The ratio of normalized primary to secondary bile acids, reflecting relative concentration changes, was significantly higher and more widely distributed in the delta senna group compared to the delta loperamide group (Figure 4D). Further exploration of the data was conducted with primary and secondary bile acids separated into individual bile acids with the five most abundant bile acids (Figure 4E). CDCA, CA, DCA and UDCA were increased in delta senna compared to delta loperamide while LCA showed no significant difference.

### 3.6 Serological Markers of Bile Acid Synthesis

**FIGURE 5:**
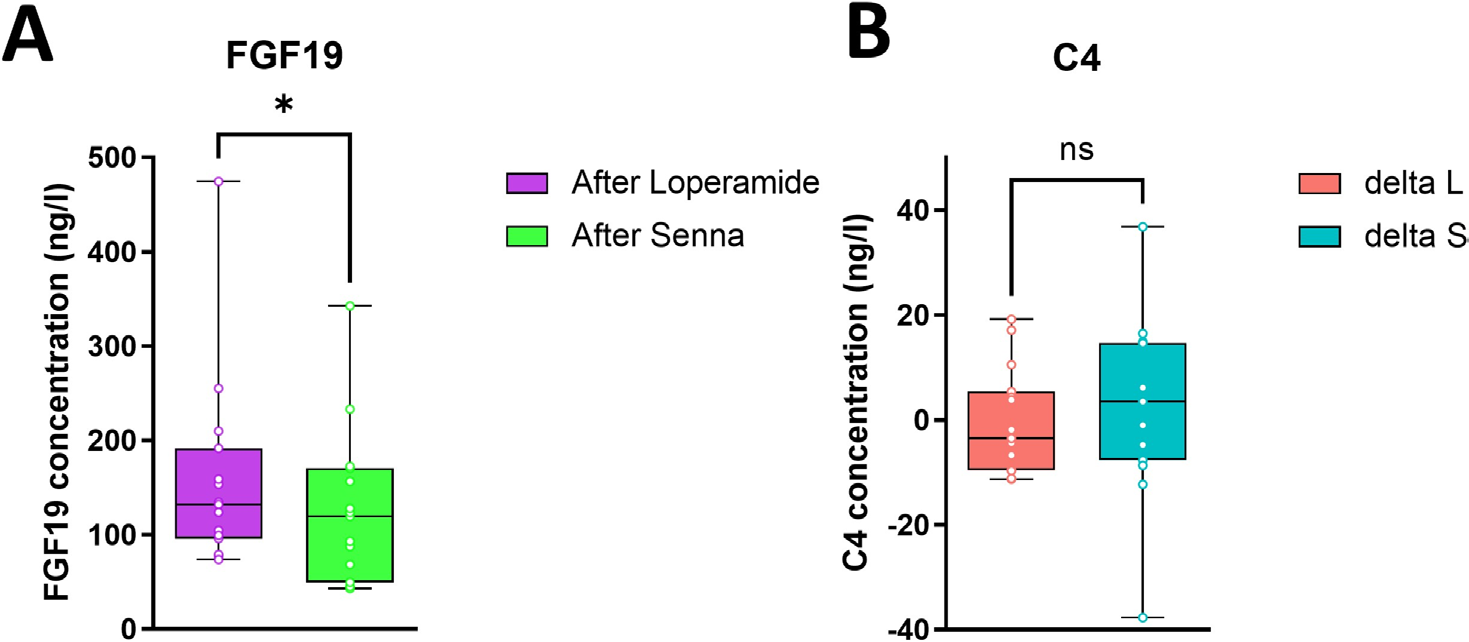
Serological markers of bile acid synthesis. (A) The concentration of fibroblast growth factor 19 (FGF19) and (B) 7α‐hydroxy‐4‐cholesten‐3‐one (C4) in serum. The box‐and‐whisker plots show the medians (horizontal lines), interquartile range (boxes), and maximum and minimum points (whiskers). Differences were assessed with the Wilcoxon test *p ≤ 0.05 and ns, not significant.

Results from the fecal bile acid suggest that a decreased gut transit time resulted in an increase of both secondary and primary fecal bile acids. To investigate if the increase of bile acids is a consequence of increased bile acid synthesis, serum levels of FGF19 and C4 (bile acid precursor) were measured. Concentrations of FGF19 and C4 in delta loperamide compared to delta senna showed no significant differences (Figure S3 and Figure 5B). However, median FGF19 levels, not normalized to baseline, were significantly higher after loperamide compared to after senna (Figure 5A). This was not the case for C4 levels; there was no significant difference after loperamide compared to after senna.

### 3.7 Correlations

An exploratory correlation study was performed to elucidate relationships between different variables affecting WGTT and bile acid metabolism, which may contribute to a deeper understanding of their physiological interactions (Figure S4). WGTT was negatively correlated with total primary and secondary bile acids (specifically CDCA, UDCA, and GCDCA), *Gemella* spp. (specifically *G. sanguinis* ), *Prevotella* spp. and *R. gnavus* , while positively correlated with species richness. Bacterial bile acid transforming genes, found in the *bai* operon, positively correlated with secondary bile acids. FGF19 and C4 were not correlated to WGTT, fecal bile acids, microbiome species or genera.

### 3.8 Baseline Assessment and Comparisons

Before the study assessment period, baseline assessment samples were collected, medians calculated, and compared (Table 1). No significant differences were observed between the two baselines for any of the measured parameters including diet assessed using DietPlan7 software.

**TABLE 1:** Comparison of baseline measurements before loperamide and senna treatment.

	WGTT	Fm	IDI	Stool weight	pBA	sBA	FGF19	α‐diversity	β‐diversity
BL‐L median	52.23	3.918	20.57	132	169.705	4039.415	145.35	87	N/A
BL‐S median	38.83	3.77	19.88	159.13	230.18	4583.745	104.22	92	N/A
*p*	0.280	0.892	0.099	0.449	0.287	0.101	0.741	0.333	0.304

*Note:* Median scores taken at baseline before loperamide (BL‐L) and baseline before senna (BL‐S) of WGTT (whole‐gut transit‐time), Fm (stool form), IDI (inter‐defecatory interval), stool weight, pBA (primary bile acids), sBA (secondary bile acids), FGF19 (fibroblast growth factor 19), and α and β diversity.

## 4 Discussion

To our knowledge, this study in healthy individuals is the first to compare changes in intestinal microbiome, bile acid composition, and metabolism after two separate pharmacological interventions, one to increase, and one to decrease WGTT. Measures of WGTT, IDI, stool form, and stool weight confirmed that loperamide and senna had the desired effects of slowing down or speeding up gut transit, increasing or reducing WGTT, respectively.

Modulating gut transit with loperamide led to a notable increase in intestinal species richness. Recent publications have consistently demonstrated a positive correlation between gut transit time and intestinal species diversity [9, 11, 33, 34]. High fecal microbial species richness has been linked to diverse diet [35] and proposed as a marker of the host health status [36, 37]. No significant difference was observed in the microbial composition (beta diversity). These findings indicate that while there are variations in species richness, interpatient microbiome differences are more pronounced than effects of our treatments. Indeed, beta diversity analysis of samples from the same individuals grouped together, regardless of whether these were pre‐ or post‐treatment, with loperamide or senna.

A significant difference in the abundance of bacterial *bsh* genes in stool samples suggest that the reduction of WGTT is associated with decrease bile acid transformation capabilities, specifically deconjugation. This is further supported by findings that senna ingestion results in higher levels of unconjugated bile acids in the stool. BSH enzymes confer a variety of benefits to the bacterial host, while the resulting unconjugated amino acid provides a source of nutrients [38, 39, 40, 41, 42, 43]. While aggregate analysis of SCFA biosynthesis genes showed no significant differences between slowed and faster gut transit, individual analysis revealed significantly elevated levels of *pta* in the slowed transit group. This suggests that while gut transit time may not broadly influence the overall synthesis of SCFAs, specific microbial pathways, such as those involving *pta*, could be selectively affected. This trend diverges from existing literature, which consistently highlights a negative correlation between gut transit time and fecal SCFA levels [41, 42, 43]. One plausible explanation for this discrepancy is differential host absorption. During slower gut transit, increased SCFA absorption by the host may result in lower fecal SCFA levels despite elevated microbial production. Further research involving direct measurement of SCFA levels in the bloodstream and feces during different WGTT will help elucidate this relationship. It is important to note that the observed differences in the abundance of bsh and the SCFA‐related gene *pta* reflect the potential production capacity of BSH and phosphate acetyltransferase, rather than directly indicating higher in situ production of these enzymes.

Although there were no changes in beta diversity as assessed via ordination, our results revealed significant changes in the abundance of several species after altering WGTT. *B. dentium*, considered beneficial for gut health, exhibited an increase following loperamide but decreased after senna. Bifidobacteria, a prevalent group of gut bacteria are known to have an important role in maintaining gut health [44, 45], and *B. dentium* is commonly present in the human microbiome. This bacterium effectively colonizes the intestinal mucus and produces SCFAs [46]. Similarly, the commensal bacterial species *C. symbiosum* was elevated after loperamide and decreased after senna. *C. symbiosum* has been shown to modify the secondary bile acid pool [47]. Little has been published regarding *Clostridium* sp. CAG 253. Alterations in WGTT likely exert selective pressures on specific microbial populations, potentially altering nutrient availability within the gut environment and leading to shifts in community dynamics. These changes may reflect a compensatory response to restore metabolic functions disrupted by loperamide or senna, as well as the utilization of substrates generated by treatment‐induced intestinal motility. While some variation was observed between the two baseline values for certain species, this was primarily in those with relatively low baseline abundance, which is expected when multiple data points are assessed. However, the effects of the interventions with senna and loperamide were substantially greater and are expressed relative to their respective baselines. These data are presented in Table S2.

Primary and secondary fecal bile acids were significantly increased in delta senna compared to delta loperamide. This can be explained by a reduced gut transit time results in less reabsorption of bile acids and consequently, this leads to an elevated amount of *de novo* primary bile acid synthesis and thus secondary bile acid biotransformation [48]. The end result is that larger quantities of primary and secondary bile acids are expelled and are detected in the stool samples as observed in our results. This rise in bile acids within the colon can initiate the onset of diarrhea, as these metabolites are known to stimulate fluid secretion [49] and defecation urge [50]. Interestingly, the primary to secondary bile acid ratio differed between delta loperamide and delta senna groups. Loperamide reduced the relative change in this ratio, suggesting increased secondary bile acid production. In contrast, senna increased the ratio and showed greater variability, indicating a more heterogeneous response in secondary bile acid metabolism. When WGTT is prolonged with loperamide, a lower proportion of primary to secondary bile acid transformation occurs compared to when gut transit is accelerated with senna. Furthermore, accelerating transit seems to have more of an effect on changing the bile acid pool than when it is slowed down with loperamide. A perturbed primary to secondary bile acid ratio has been used as a diagnostic marker for BAD [51]. Future experiments could benefit from analyzing additional forms of bile acids, including isomers, alongside microbiota data to enhance understanding of bacterial metabolism.

Decreased gut transit time, after senna compared to loperamide, results in a decrease of the hormone FGF19 (which acts to inhibit bile acid synthesis). Potentially, more rapid transit through the ileum reduces bile acid reabsorption and the subsequent stimulation of FGF19 transcription, affecting the quantity released. This offers insight into the elevation of primary and subsequently secondary bile acids observed. However, a significant disparity was not observed in the concentration of FGF19 and C4 between the delta loperamide group and the delta senna group. The hormone FGF19 and metabolite C4 are highly variable and affected by many factors. Further research may help elucidate the underlying mechanisms driving these observations.

Negative correlations exist between the WGTT and stool bile acids, key species of the gut microbiota, and the *bai* genes involved in bile acid transformation. A shorter gut transit time reflects a higher concentration of primary and secondary bile acids (specifically CDCA, UDCA, and GCDCA), more abundant *Gemella* (specifically *G. sanguinis* ) *Prevotella* spp. and *R. gnavus* , while the species richness is significantly decreased. The negative correlation between bile acids and the WGTT is consistent with our observations, as illustrated by significantly increased primary and secondary bile acids in delta senna compared to delta loperamide. The natural potent farnesoid X receptor (FXR) agonist, CDCA, exhibited a negative correlation with WGTT. This finding, when considered independently, suggests that CDCA may lead to a decrease in bile acid synthesis when WGTT is reduced as bile acids regulate their own expression. However, both UDCA and GCDCA are also negatively associated with WGTT and have been reported in literature to effectively inhibit FXR activation induced by CDCA [52]. The aforementioned findings provide a logical explanation for the observed elevation of: FGF19 hormone, primary and secondary bile acids, stool form, and stool weight in volunteers with reduced WGTT. Our findings indicate that by changing WGTT, there was a notable change in the abundance of specific bacterial species. Of these, *R. gnavus* and potentially *G. sanguinis* could be used as a marker for early onset dysbiosis caused by diarrheal episodes.

The absence of significant differences between the baseline assessment groups is noteworthy, as it suggests that the gut microbiome can return to its original state within just 16 days after discontinuing loperamide or senna treatment, highlighting the gut's ability to recover from short‐term disturbances. There is considerable diversity among the samples as individuals possess their own unique bile acid and microbiota profile. This observation supports the rationale for personalized medicine, as it highlights the potential for individual differences in response to treatment.

Several limitations should be considered. Long‐term impacts of altered WGTT were not assessed, as the study involved short‐term administration of loperamide and senna. However, significant alterations in bile acid and microbiota profiles were nonetheless observed. Additionally, the study was conducted on healthy individuals, so findings might not directly translate to patients with gastrointestinal disorders. Nonetheless, the results provide valuable insights into WGTT's influence on gut homeostasis. Senna may affect gut secretion and permeability, potentially altering microbiota composition and bile acid profiles independently of transit time changes [53, 54]. Finally, the antimicrobial activity of senna [55] may have influenced the observed microbiome and bile acid changes independently of its laxative effects. Though, our confidence in using these drugs to modify WGTT and assess their effects is supported by our prior publications [41, 43].

## 5 Conclusion

These findings suggest some previously established microbiome‐disease correlations could reflect altered gut transit. Previous research has noted alterations in the gut microbiota, levels of bile acids and WGTT in various GI disorders. However, the causal relationship between these changes has not been established. This study in healthy individuals provides strong evidence that changes in WGTT are a contributing factor to disruptions in the intestinal microbiota and bile acid balance. Our findings strongly suggest that gut transit time should be considered a crucial factor in future microbiome research, to obtain more accurate and comprehensive results.

## Author Contributions

**Evette B. M. Hillman:** conceptualization: supporting; methodology: equal; formal analysis: lead; investigation: equal; data curation: lead; writing – original draft; visualization: lead; project administration: equal. **Maximilian Baumgartner:** formal analysis: supporting; data curation: supporting; writing – review and editing: equal; visualization: supporting. **Danielle Carson:** formal analysis: supporting; data curation: supporting; writing – review and editing: equal; visualization: supporting; supervision: equal. **Gregory C. A. Amos:** writing – review and editing: equal; supervision: equal. **Imad Wazir:** investigation: equal. **Haider A. Khan:** investigation: equal. **Malik A. Khan:** investigation: equal. **Sjoerd Rijpkema:** writing – review and editing: equal; supervision: equal. **Julian R. F. Walters:** writing – review and editing: equal; supervision: equal. **Elizabeth M. H. Wellington:** resources: supporting; writing – review and editing: equal; supervision: equal. **Ramesh Arasaradnam:** writing – review and editing: equal; supervision: equal. **Stephen J. Lewis:** conceptualization: lead; methodology: equal; investigation: equal; resources: lead; writing – review and editing: equal; supervision: equal; project administration: equal.

## Disclosure

Institutional review board statement: The views expressed in the publication are those of the author(s) and not necessarily those of the NHS, the NIHR, the Department of Health, “arms” length bodies or other government departments.

## Ethics Statement

The study was reviewed and approved by the ethics committee of the NHS Health Research Authority and Health and Care Research Wales (18/NW/0346). The study was conducted in accordance with the ethical principles expressed by Research Ethics Committee (REC).

## Conflicts of Interest

The authors declare no conflicts of interest.

## Supporting information


**Table S1.** Demographic and baseline characteristics of volunteers participating in the cross‐over study.
**Table S2**. Significant Bacterial Species Identified Between Post‐Drug and Baseline Conditions with Associated Metrics from DESeq2 Analysis baseMean (mean of normalized counts for baseline samples), log_2_FC (log_2_ fold change), lfcSE (the standard error estimate for the log_2_ fold change estimate), stat (Wald statistic), *p*value (Wald test *p*‐value), *p*adj (Benjamini‐Hochberg adjusted *p*‐values), Delta (δ‐Loperamide and δ‐Senna) and direction of change.
**Table S3**. Bile acids in their conjugated and sulfated form. Wilcoxon paired statistical test was used to obtain *p*‐values list. CA‐3S, cholic acid 3‐sulfate; CDCA‐3S, chenodeoxycholic; DCA‐3S, deoxycholic acid 3‐sulfate; GCA, glycocholic acid; GCDCA, glycochenodeoxycholic acid; GDCA, glycodeoxycholic acid; GLCA, glyolithocholic acid; GLCA‐3S, glyolithocholic acid 3‐sulfate; GUDCA, glycoursodeoxycholic acid; HCA, hyocholic acid; LCA‐3S, lithocholic acid 3‐sulfate; TCA, taurocholic acid; TCDCA, taurochenodeoxycholic acid; TDCA, taurodeoxycholic acid; TLCA, taurolithocholic acid; TLCA‐3S, taurolithocholic acid 3‐sulfate; TUDCA, tauroursodeoxycholic acid; UDCA‐3S, ursodeoxycholic acid 3‐sulfate.
**Figure S1**. Effects of loperamide or senna on markers of gut transit and bowel function. (A) WGTT (whole‐gut transit‐time), (B) IDI (inter‐defecatory interval) (C) Fm (stool form), and (D) stool weight per day before and after (delta) taking loperamide (*n* = 16) and senna (*n* = 18). The box‐and‐whisker plots show the medians (horizontal lines), interquartile range (boxes), and maximum and minimum points (whiskers). *p*‐values calculated Wilcoxon test *p ≤ 0.05 and ***p ≤ 0.001.
**Figure S2**. Beta‐diversity of sample groups. Bacterial species clustering by sample groups: Baseline_L (red) *n* = 16, Loperamide (blue) *n* = 16, Baseline_S (green) *n* = 18 and senna (purple) *n* = 18. Nonmetric multidimensional scaling (NMDS) plot with Bray–Curtis dissimilarity distances. Plot ellipses represent the 95% confidence regions for group clusters. *p*‐values calculated unweighted UniFrac test *p* ≤ 0.99.
**Figure S3**. The concentration of fibroblast growth factor 19 (FGF19) in serum before and after (delta) taking loperamide and senna drug. The box‐and‐whisker plots show the medians (horizontal lines), interquartile range (boxes), and maximum and minimum points (whiskers). Differences were assessed with the Wilcoxon test. ns, not significant.
**Figure S4**. Correlation matrices of WGTT (whole gut transit time), bile acids, species richness, species genus, species and bile acid transforming genes. Spearman's correlation coefficient to determine the color (red = positive correlation and blue = negative correlation). Significant (*p* ≤ 0.05) adjusted *p*‐values were calculated using the BH method are indicated with an asterisk. *N* = 68.

## Data Availability

Metagenomic sequencing files will be made publicly available on NCBI Nucleotide database. Sequence Read Archive (SRA) submission: SUB14651183. Impact of Transit Time on Microbiome and Bile Acids, Aug 07 '24.
