# Supplementary material for: Changing Gastrointestinal Transit Time Alters Microbiome Composition and Bile Acid Metabolism: A Cross‐Over Study in Healthy Volunteers
Source: Neurogastroenterol Motil. 2025 May 20;37(10):e70075. doi: 10.1111/nmo.70075 (PMC12435802; doi:10.1111/nmo.70075)
Supplement: Supplementary file 1 — Table S1. Demographic and baseline characteristics of volunteers participating in the cross‐over study. Table S2. Significant Bacterial Species Identified Between Post‐Drug and Baseline Conditions with Associated Metrics from DESeq2 Analysis baseMean (mean of normalized counts for baseline samples), log2FC (log2 fold change), lfcSE (the standard error estimate for the log2 fold change estimate), stat (Wald statistic), pvalue (Wald test p‐value), padj (Benjamini‐Hochberg adjusted p‐values), Delta (δ‐Loperamide and δ‐Senna) and direction of change. Table S3. Bile acids in their conjugated and sulfated form. Wilcoxon paired statistical test was used to obtain p‐values list. CA‐3S, cholic acid 3‐sulfate; CDCA‐3S, chenodeoxycholic; DCA‐3S, deoxycholic acid 3‐sulfate; GCA, glycocholic acid; GCDCA, glycochenodeoxycholic acid; GDCA, glycodeoxycholic acid; GLCA, glyolithocholic acid; GLCA‐3S, glyolithocholic acid 3‐sulfate; GUDCA, glycoursodeoxycholic acid; HCA, hyocholic acid; LCA‐3S, lithocholic acid 3‐sulfate; TCA, taurocholic acid; TCDCA, taurochenodeoxycholic acid; TDCA, taurodeoxycholic acid; TLCA, taurolithocholic acid; TLCA‐3S, taurolithocholic acid 3‐sulfate; TUDCA, tauroursodeoxycholic acid; UDCA‐3S, ursodeoxycholic acid 3‐sulfate. Figure S1. Effects of loperamide or senna on markers of gut transit and bowel function. (A) WGTT (whole‐gut transit‐time), (B) IDI (inter‐defecatory interval) (C) Fm (stool form), and (D) stool weight per day before and after (delta) taking loperamide (n = 16) and senna (n = 18). The box‐and‐whisker plots show the medians (horizontal lines), interquartile range (boxes), and maximum and minimum points (whiskers). p‐values calculated Wilcoxon test *p ≤ 0.05 and ***p ≤ 0.001. Figure S2. Beta‐diversity of sample groups. Bacterial species clustering by sample groups: Baseline_L (red) n = 16, Loperamide (blue) n = 16, Baseline_S (green) n = 18 and senna (purple) n = 18. Nonmetric multidimensional scaling (NMDS) plot with Bray–Curtis dissimilar [file NMO-37-e70075-s001.docx]

**Supplementary material**

**Supplementary Table 1**: Demographic and baseline characteristics of volunteers participating in the cross-over study

| Gender (M/F) | Age | Weight | Height | BMI | Gallstones | Norm Bowel Freq |
| --- | --- | --- | --- | --- | --- | --- |
| M | 54 | 80 | 1.79 | 24.97 | 0 | 1 |
| M | 36 | 88 | 1.73 | 29.40 | 0 | 2 |
| M | 28 | 84 | 1.72 | 28.39 | 0 | 1 |
| M | 28 | 84 | 1.91 | 23.03 | 0 | 1 |
| M | 35 | 70 | 1.83 | 20.90 | 0 | 1 |
| M | 29 | 80 | 1.77 | 25.54 | 0 | 1.5 |
| M | 24 | 75 | 1.8 | 23.15 | 0 | 2.5 |
| F | 53 | 63 | 1.64 | 23.42 | 0 | 1.5 |
| M | 28 | 68 | 1.67 | 24.38 | 0 | 1 |
| F | 42 | 78 | 1.68 | 27.64 | 0 | 2.5 |
| M | 28 | 94 | 1.77 | 30.00 | 0 | 1.5 |
| M | 24 | 92.3 | 1.83 | 27.56 | 0 | 1 |
| F | 64 | 56.5 | 1.62 | 21.53 | 0 | 1 |
| F | 49 | 59 | 1.7 | 20.42 | 0 | 1.5 |
| M | 31 | 84 | 1.78 | 26.51 | 0 | 2 |
| M | 53 | 85 | 1.8 | 26.23 | 0 | 3 |
| F | 67 | 62 | 1.52 | 26.84 | 0 | 1 |
| F | 33 | 73.2 | 1.62 | 27.89 | 0 | 1 |

This table summarizes the demographic and baseline characteristics of the study participants, including gender distribution, age, weight, height, body mass index (BMI), presence of gallstones, and self-reported normal bowel movement frequency.


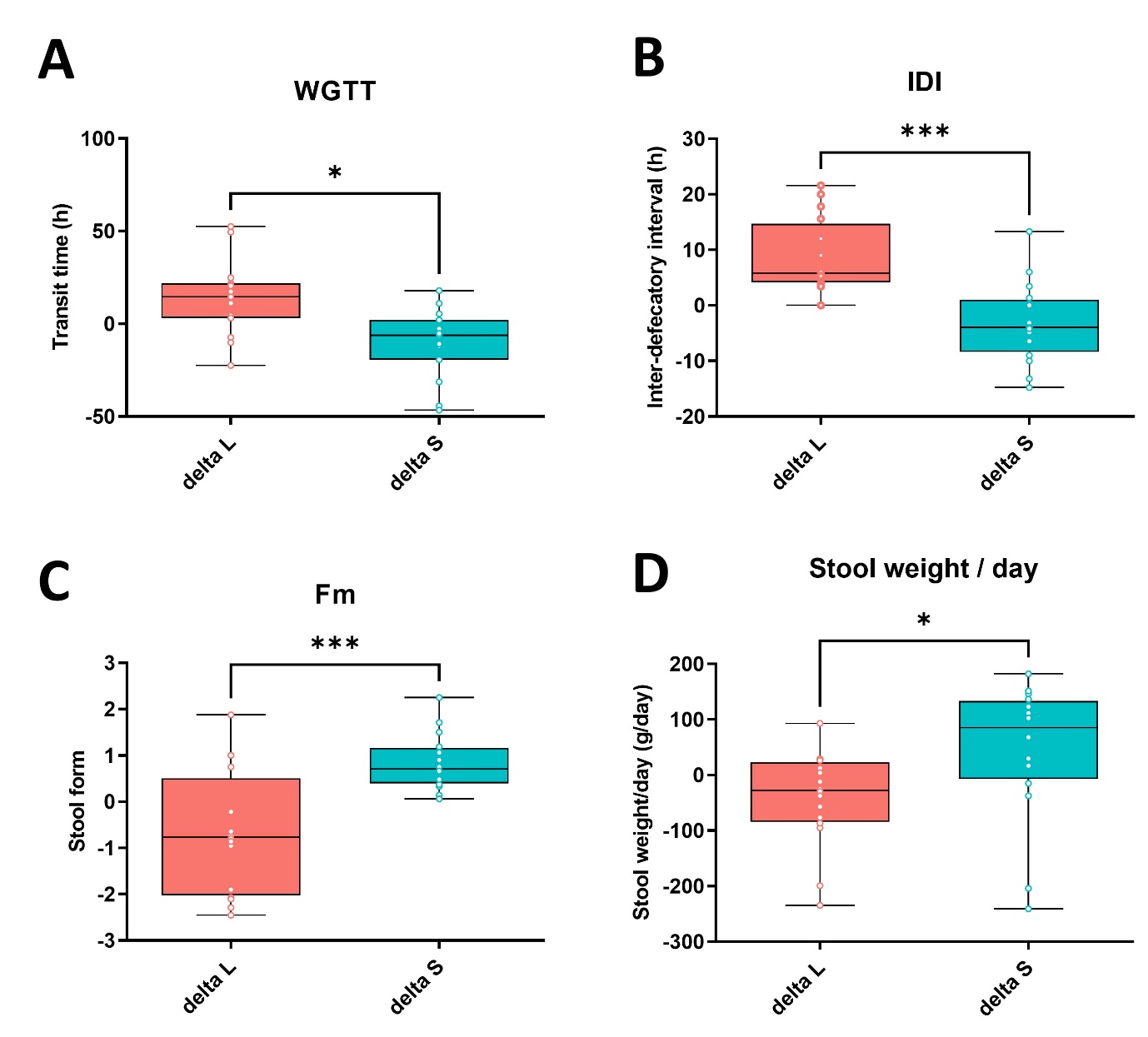


**Supplementary Figure 1: Effects of loperamide or senna on markers of gut transit and bowel function.** (**A**) WGTT (whole-gut transit-time), (**B**) IDI (inter-defecatory interval) (**C**) Fm (stool form), and (**D**) stool weight per day before and after (delta) taking loperamide (n=16) and senna (n=18). The box-and-whisker plots show the medians (horizontal lines), interquartile range (boxes), and maximum and minimum points (whiskers). P-values calculated Wilcoxon test **p≤0.05* and *** *p ≤ 0.001*


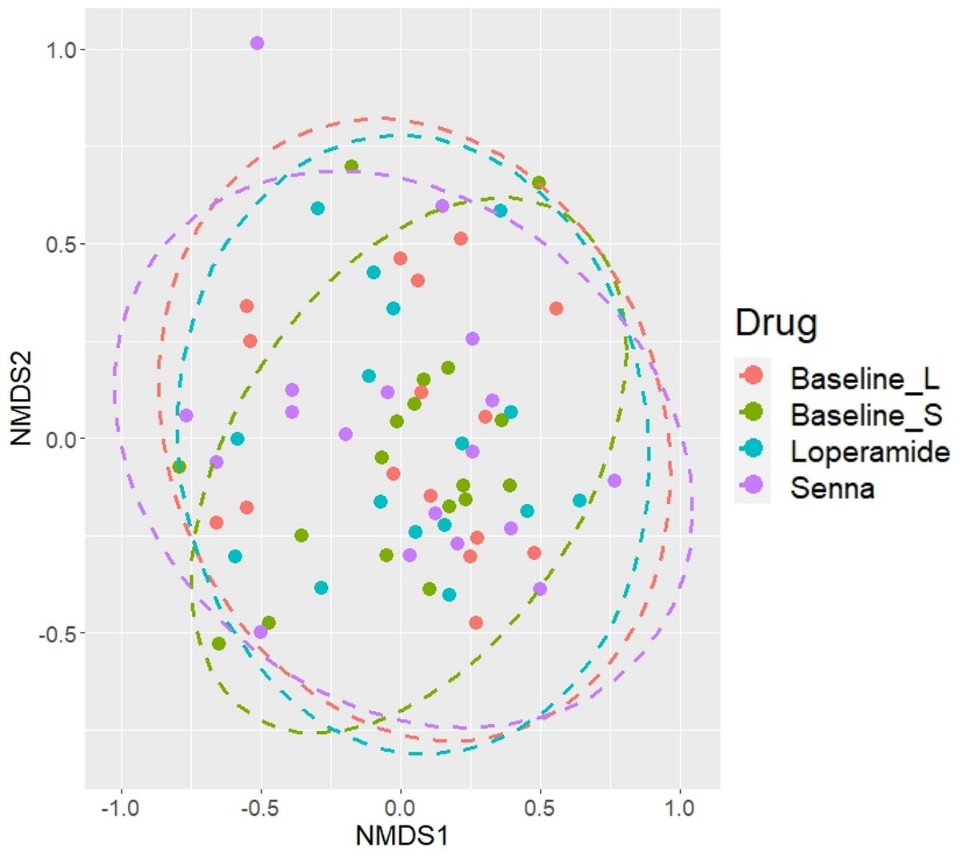


**Supplementary Figure 2**: **Beta-diversity of sample groups**. Bacterial species clustering by sample groups: Baseline_L (red) n=16, Loperamide (blue) n=16, Baseline_S (green) n=18 and senna (purple) n=18. Non-metric multidimensional scaling (NMDS) plot with Bray-Curtis dissimilarity distances. Plot ellipses represent the 95% confidence regions for group clusters. P-values calculated unweighted UniFrac test *p≤0.99*

**Supplementary Table 2**: Significant Bacterial Species Identified Between Post-Drug and Baseline Conditions with Associated Metrics from DESeq2 Analysis baseMean (mean of normalised counts for baseline samples), log2FC (log_2_ fold change), lfcSE (the standard error estimate for the log2 fold change estimate), stat (Wald statistic), pvalue (Wald test p-value), padj (Benjamini-Hochberg adjusted p-values), Delta (δ-Loperamide and δ-Senna) and direction of change.

| **Species** | **baseMean** | **log2FC** | **lfcSE** | **stat** | **pvalue** | **padj** | **Delta** | **Change** |
| --- | --- | --- | --- | --- | --- | --- | --- | --- |
| *Allisonella_histaminiformans* | 99.53 | -26.95 | 2.93 | -9.20 | *3.72E-20* | *7.93E-19* | δ-Loperamide | down |
| *Anaerotruncus_sp_CAG_528* | 4414.68 | 30.00 | 2.93 | 10.24 | *1.33E-24* | *2.56E-22* | δ-Loperamide | up |
| *Bacteroides_sp_CAG_144* | 2650.53 | -6.61 | 2.89 | -2.29 | *2.20E-02* | *1.92E-01* | δ-Loperamide | down |
| *Bifidobacterium_angulatum* | 913.39 | 28.15 | 2.93 | 9.61 | *7.55E-22* | *4.83E-20* | δ-Loperamide | up |
| *Bifidobacterium_dentium* | 433.89 | 28.00 | 2.93 | 9.56 | *1.21E-21* | *5.82E-20* | δ-Loperamide | up |
| *Butyrivibrio_sp_CAG_318* | 235.71 | 27.15 | 2.93 | 9.27 | *1.94E-20* | *5.33E-19* | δ-Loperamide | up |
| *Clostridium_aldenense* | 44.95 | -25.86 | 2.93 | -8.82 | *1.11E-18* | *1.63E-17* | δ-Loperamide | down |
| *Clostridium_sp_CAG_253* | 31.32 | -25.37 | 2.93 | -8.66 | *4.90E-18* | *6.72E-17* | δ-Loperamide | down |
| *Clostridium_symbiosum* | 4.56 | 21.64 | 2.93 | 7.38 | *1.62E-13* | *1.55E-12* | δ-Loperamide | up |
| *Desulfovibrionaceae_bacterium* | 175.00 | -27.70 | 2.93 | -9.45 | *3.28E-21* | *1.26E-19* | δ-Loperamide | down |
| *Faecalitalea_cylindroides* | 149.60 | 26.51 | 2.93 | 9.05 | *1.46E-19* | *2.55E-18* | δ-Loperamide | up |
| *Gemella_sanguinis* | 7.53 | -23.43 | 2.93 | -7.99 | *1.34E-15* | *1.43E-14* | δ-Loperamide | down |
| *Lactobacillus_paragasseri* | 95.84 | -26.90 | 2.93 | -9.18 | *4.36E-20* | *8.38E-19* | δ-Loperamide | down |
| *Lactococcus_lactis* | 5.65 | 21.94 | 2.93 | 7.48 | *7.36E-14* | *7.44E-13* | δ-Loperamide | up |
| *Megamonas_funiformis* | 59.37 | 25.22 | 2.93 | 8.61 | *7.50E-18* | *9.60E-17* | δ-Loperamide | up |
| *Olsenella_scatoligenes* | 32.22 | 24.37 | 2.93 | 8.31 | *9.19E-17* | *1.04E-15* | δ-Loperamide | up |
| *Prevotella_sp_CAG_1092* | 1016.82 | 29.05 | 2.93 | 9.92 | *3.57E-23* | *3.43E-21* | δ-Loperamide | up |
| *Streptococcus_sanguinis* | 106.93 | -27.03 | 2.93 | -9.23 | *2.82E-20* | *6.76E-19* | δ-Loperamide | down |
| *Streptococcus_sp_A12* | 55.52 | -26.15 | 2.93 | -8.92 | *4.51E-19* | *7.22E-18* | δ-Loperamide | down |
| *Streptococcus_vestibularis* | 283.80 | 27.41 | 2.93 | 9.35 | *8.47E-21* | *2.71E-19* | δ-Loperamide | up |
| *Turicibacter_sanguinis* | 1771.08 | 6.61 | 2.27 | 2.91 | *3.59E-03* | *3.28E-02* | δ-Loperamide | up |
| *Turicimonas_muris* | 45.08 | 24.84 | 2.93 | 8.48 | *2.35E-17* | *2.81E-16* | δ-Loperamide | up |
| *Actinomyces_sp_ICM47* | 65.05 | 25.51 | 2.93 | 8.72 | *2.77E-18* | *7.32E-17* | δ-Senna | up |
| *Actinomyces_sp_S6_Spd3* | 10.31 | 22.94 | 2.93 | 7.84 | *4.58E-15* | *6.87E-14* | δ-Senna | up |
| *Bacteroides_finegoldii* | 503.96 | -29.10 | 2.93 | -9.95 | *2.53E-23* | *1.56E-21* | δ-Senna | down |
| *Bacteroides_sp_CAG_530* | 1938.69 | 30.00 | 2.93 | 10.26 | *1.11E-24* | *1.03E-22* | δ-Senna | up |
| *Bifidobacterium_dentium* | 6.47 | -23.15 | 2.93 | -7.91 | *2.60E-15* | *4.37E-14* | δ-Senna | down |
| *Butyrivibrio_crossotus* | 2439.42 | 30.00 | 2.93 | 10.26 | *1.11E-24* | *1.03E-22* | δ-Senna | up |
| *Clostridium_sp_CAG_253* | 219.36 | 27.21 | 2.93 | 9.30 | *1.39E-20* | *6.45E-19* | δ-Senna | up |
| *Clostridium_symbiosum* | 44.38 | -25.78 | 2.93 | -8.81 | *1.24E-18* | *3.83E-17* | δ-Senna | down |
| *Eubacterium_rectale* | 1340231.15 | 1.93 | 0.92 | 2.10 | *3.61E-02* | *4.18E-01* | δ-Senna | up |
| *Firmicutes_bacterium_CAG_94* | 55.04 | -24.44 | 2.93 | -8.35 | *6.58E-17* | *1.22E-15* | δ-Senna | down |
| *Massiliomicrobiota_timonensis* | 117.94 | -6.73 | 2.90 | -2.32 | *2.02E-02* | *2.64E-01* | δ-Senna | down |
| *Mitsuokella_multacida* | 5.47 | -22.92 | 2.93 | -7.83 | *4.82E-15* | *6.87E-14* | δ-Senna | down |
| *Oscillibacter_sp_PC13* | 64.69 | 25.30 | 2.93 | 8.65 | *5.17E-18* | *1.20E-16* | δ-Senna | up |
| *Ruminococcaceae_bacterium_D5* | 124.38 | 26.42 | 2.93 | 9.03 | *1.70E-19* | *6.30E-18* | δ-Senna | up |
| *Ruminococcus_gnavus* | 1281.82 | 6.33 | 2.75 | 2.30 | *2.14E-02* | *2.64E-01* | δ-Senna | up |
| *Ruminococcus_sp_CAG_488* | 49.38 | 25.12 | 2.93 | 8.59 | *8.87E-18* | *1.82E-16* | δ-Senna | up |
| *Streptococcus_australis* | 144.77 | 5.86 | 2.89 | 2.03 | *4.25E-02* | *4.62E-01* | δ-Senna | up |

**Supplementary Table 3**: Bile acids in their conjugated and sulfated form. Wilcoxons paired statistical test was used to obtain p-values list. TUDCA, tauroursodeoxycholic acid; TCDCA, taurochenodeoxycholic acid; TDCA, taurodeoxycholic acid; TCA, taurocholic acid; TLCA-3S, taurolithocholic acid 3-sulfate; GLCA-3S, glyolithocholic acid 3-sulfate; UDCA-3S, ursodeoxycholic acid 3-sulfate; CDCA-3S, chenodeoxycholic; DCA-3S, deoxycholic acid 3-sulfate; LCA-3S, lithocholic acid 3-sulfate; CA-3S, cholic acid 3-sulfate; GLCA, glyolithocholic acid; GUDCA, glycoursodeoxycholic acid; GCDCA, glycochenodeoxycholic acid; GDCA, glycodeoxycholic acid; GCA, glycocholic acid; TLCA, taurolithocholic acid; HCA, hyocholic acid.

*next to the significant values

|  | **Median** | | **P value** |
| --- | --- | --- | --- |
|  | **delta L** | **delta S** |  |
| **TUDCA** | 0.000 | 0.000 | *0.125* |
| **TCDCA*** | -4.102 | 3.493 | *0.004* |
| **TDCA*** | -0.768 | 1.676 | *0.048* |
| **TCA*** | -4.080 | 3.699 | *0.010* |
| **TLCA-3S** | 0.000 | 0.000 | *0.125* |
| **GLCA-3S** | 0.000 | 0.000 | *0.168* |
| **UDCA-3S** | 0.000 | 0.000 | *0.219* |
| **CDCA-3S** | 0.000 | 0.000 | *0.688* |
| **DCA-3S** | 0.000 | 0.000 | *0.638* |
| **LCA-3S*** | 0.000 | 12.187 | *0.008* |
| **CA-3S** | 0.000 | 0.000 | *>0.999* |
| **GLCA** | 0.000 | 0.000 | *0.500* |
| **GUDCA** | 0.000 | 0.000 | *0.123* |
| **GCDCA*** | -9.864 | 18.426 | *0.008* |
| **GDCA*** | -4.721 | 20.156 | *0.000* |
| **GCA** | -3.846 | 10.694 | *0.064* |
| **TLCA** | 0.000 | 0.000 | *0.813* |
| **LCA** | -412.524 | 328.958 | *0.359* |
| **UDCA*** | 0.550 | 14.747 | *0.002* |
| **CDCA*** | -5.049 | 50.679 | *0.005* |
| **DCA*** | -604.306 | 1336.234 | *0.018* |
| **CA*** | -17.292 | 55.324 | *0.007* |
| **HCA*** | 0.000 | 0.000 | *0.020* |

**Supplementary Figure 3**: **The concentration of fibroblast growth factor 19 (FGF19)** in serum before and after (delta) taking loperamide and senna drug. The box-and-whisker plots show the medians (horizontal lines), interquartile range (boxes), and maximum and minimum points (whiskers). Differences were assessed with the Wilcoxons test. ns=not significant


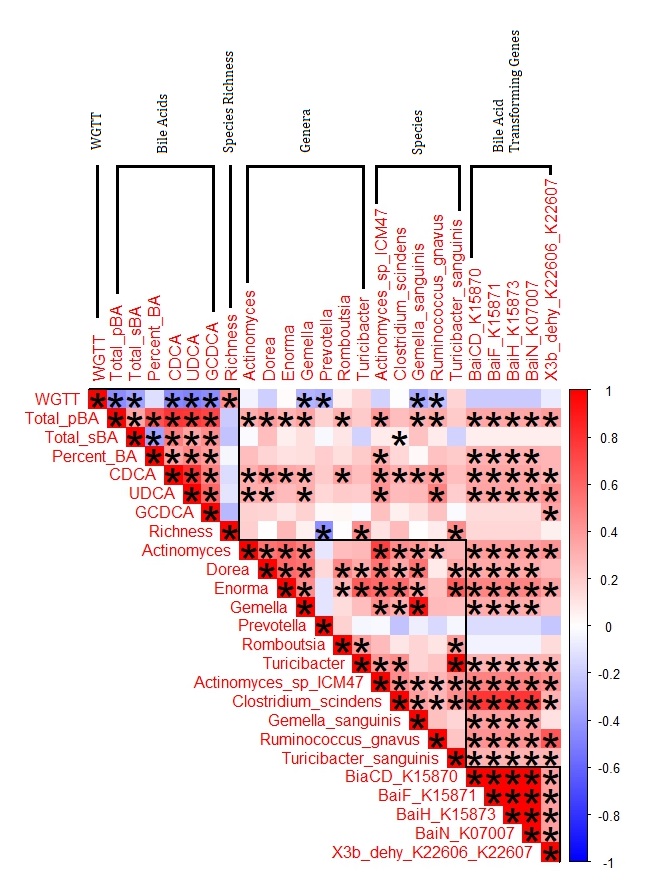


**Figure** Error! No text of specified style in document.**.1**: Correlation matrices of whole gut transit time, bile acids, species

**Supplementary Figure 4**: Correlation matrices of WGTT (whole gut transit time), bile acids, species richness, species genus, species and bile acid transforming genes. Spearman’s correlation coefficient to determine the colour (red= positive correlation and blue= negative correlation). Significant (p ≤.05) adjusted p-values were calculated using the BH method are indicated with an asterisk. N=68
